# Effects of Severe Sleep Disruption on the Synaptic Ultrastructure of Young Mice

**DOI:** 10.1523/ENEURO.0077-21.2021

**Published:** 2021-07-13

**Authors:** Hirotaka Nagai, Luisa de Vivo, William Marshall, Giulio Tononi, Chiara Cirelli

**Affiliations:** 1 Department of Psychiatry, University of Wisconsin-Madison, Madison, WI 53719; 2 Department of Mathematics and Statistics, Brock University, St. Catharines, Ontario L2S 3A1, Canada

## Abstract

There is molecular, electrophysiological, and ultrastructural evidence that a net increase in synaptic strength occurs in many brain circuits during spontaneous wake (SW) or short sleep deprivation, reflecting ongoing learning. Sleep leads instead to a broad but selective weakening of many forebrain synapses, thus preventing synaptic saturation and decreasing the energy cost of synaptic activity. Whether synaptic potentiation can persist or further increase after long sleep deprivation is unknown. Whether synaptic renormalization can occur during chronic sleep restriction (CSR) is also unknown. Here, we addressed these questions by measuring an established ultrastructural measure of synaptic strength, the axon-spine interface (ASI), in the primary motor cortex (M1) of (1) one-month-old adolescent mice CSR using a paradigm that decreases NREM and REM sleep by two/thirds; (2) in two-week-old mouse pups sleep deprived for 15 h, or allowed afterward to recover for 16 h. Both groups were compared with mice of the same age that were asleep or awake for a few hours (both sexes). The ASI size of CSR mice (*n* = 3) was comparable to that measured after SW or short sleep deprivation and larger than after sleep (*n* = 4/group). In pups, the ASI size increased after short sleep loss (*n* = 3) relative to sleep (*n* = 4), fell below sleep levels after long sleep deprivation (*n* = 4), and remained low after recovery (*n* = 3). Long sleep deprived pups also lost some weight. These results suggest that (1) severe sleep restriction is incompatible with synaptic renormalization; (2) very young mice cannot maintain high synaptic strength during prolonged wake.

## Significance Statement

The strength of many excitatory synapses increases during wake and decreases during sleep. However, because strong synapses require more energy, synaptic potentiation may be difficult to maintain when wake is enforced well beyond its physiological duration, especially in young animals whose brain is growing. Moreover, because synaptic weakening ultimately requires structural changes and the endocytosis of excitatory receptors, limited and disrupted sleep may not be compatible with synaptic renormalization. We confirmed the first prediction in two-week-old pups kept awake for 15 h, and the second prediction in one-month-old mice severely sleep restricted for 4 d.

## Introduction

We recently used serial block-face scanning electron microscopy (SBEM) to obtain high-resolution measurements of the axon-spine interface (ASI), the direct area of contact between presynapse and postsynapse, in thousands of excitatory glutamatergic synapses of the mouse brain (Cirelli and Tononi, 2020). The ASI is a morphological measure of synaptic strength whose size correlates with the amplitude of the evoked unitary excitatory postsynaptic currents and with the area of the postsynaptic density (Cheetham et al., 2014). Like the latter, the ASI increases with synaptic potentiation (Desmond and Levy, 1988; Buchs and Muller, 1996; Fukazawa et al., 2003; Cheetham et al., 2014). The goal of our experiments was to use the ASI to test the synaptic homeostasis hypothesis of sleep function (Tononi and Cirelli, 2014, 2020). According to this hypothesis, ongoing learning during wake leads to a net increase in synaptic strength in many brain circuits, and sleep is needed to renormalize synaptic weights to save energy, avoid saturation, and benefit memory consolidation and integration. So far, this hypothesis has been tested in the superficial layers of primary motor and sensory cortex (de Vivo et al., 2017), as well as in the stratum radiatum of the CA1 region of the hippocampus (Spano et al., 2019). In all cases, we found that the size of the ASI was on average smaller in one-month-old adolescent mice that slept for 6–7 h during the light phase, compared to mice that during the same time of day were kept awake by exposure to novel objects. In the primary motor and sensory cortex the ASI was also larger after 6–7 h of spontaneous wake (SW) during the dark phase compared to after sleep, and the average size of the ASI was similar in mice awake at night and in mice sleep deprived during the day (de Vivo et al., 2017). We also found that, in primary motor cortex (M1), the ASI was larger in two-week-old mouse pups kept awake for 4–5 h during the day as compared to siblings allowed to sleep during the same time window (de Vivo et al., 2019). Together, these findings show that the ASI of cortical synapses is larger after wake than after sleep independent of circadian time, both in early development and during adolescence, in line with the synaptic homeostasis hypothesis (Tononi and Cirelli, 2014, 2020).

In the experiments performed so far, mice were spontaneously awake or totally sleep deprived for only a few hours. It is not known how more severe acute sleep loss, or chronic sleep restriction (CSR), affects the ASI. To address these questions, we measured the size of the ASI in two sets of animals: (1) one-month-old adolescent mice whose sleep was restricted to a third of the normal daily amount for 4–5 d; (2) two-week-old mouse pups forced to be awake for up to 15 consecutive hours. To further assess the effects of severe sleep loss, in the same groups of mice we also analyzed several other features of the organelles inside the spines.

## Materials and Methods

### Animals

We used homozygous B6.Cg-Tg(Thy1-YFP)16Jrs/J transgenic mice (YFP-H, The Jackson Laboratory; IMSR catalog #JAX:003709, 78RRID:IMSR_JAX:003709) that express yellow fluorescent protein (YFP) in a subset of cortical pyramidal neurons (Feng et al., 2000). The developmental changes in the sleep/wake pattern of these mice have been characterized in previous studies (Nelson et al., 2013; de Vivo et al., 2019), and this strain was used in previous SBEM experiments (de Vivo et al., 2017, 2019; Spano et al., 2019). All animal facilities were reviewed and approved by the Institutional Animal Care and Use Committee (IACUC) of the University of Wisconsin-Madison, and were inspected and accredited by Association for Assessment and Accreditation of Laboratory Animal Care (AAALAC).

### Experimental groups for SBEM studies

#### Postnatal day (P)13 mice

B6.Cg-Tg(Thy1-YFP)16Jrs/J mice were inspected every day for new pups. P0 was defined as the day of birth. The entire litters (both sexes) and the dams were housed together in environmentally controlled recording chambers with free access to food and water (12/12 h light/dark cycle; lights on at 8 A.M.). At the beginning of each dark period a running wheel and one to two novel objects were introduced in each recording chamber, to ensure enriched housing conditions and facilitate the light/dark entrainment of the rest/activity cycle. Running wheels and novel objects were removed during the light period. The day of the experiment (P13), at the onset of the light phase, mice were left undisturbed for 4–5 h [sleep (S), four mice], or sleep deprived as much as possible for 4–5 h [extended waking (EW)4, three mice], or for 15–16 h (EW15, four mice) using a combination of different stimuli including novel objects and gentle handling, during which the cage was gently tilted, the pups were touched with a soft brush, or they were gently poked by the experimenter. These stimuli were applied any time the pups attempted to fall asleep. Another group was sleep deprived for ∼15 h and then allowed to sleep *ad libitum* for 15–16 h (Rec, three mice). S and EW4 pups came from the same litter, and so did EW15 and recovery (Rec) mice. The exact duration of sleep or EW varied because it took ∼1 h to perfuse the entire litter, comprising seven to eight pups. In a previous study (de Vivo et al., 2019), we performed behavioral sleep/wake scoring in 30 s epochs in pairs of P13 Thy1-YFP siblings housed with the dam and found that during the light phase these pups spend roughly 50% of each hour asleep, independent of circadian time. In that study (de Vivo et al., 2019), we also performed SBEM experiments in pups allowed to sleep or sleep deprived for 4–5 h (de Vivo et al., 2019), but the results reported here are from different sets of S and EW4 animals that were collected together with the EW15 and Rec mice. As previously reported (de Vivo et al., 2019), even after the first few hours of forced wake, pups responded to the stimuli much less than older mice, and it was impossible to keep them fully awake for extended periods of time. For this reason, the long sleep deprivation experiment, which was planned to last for 24 h, was terminated after 15–16 h. In the previous (de Vivo et al., 2019) and the current SBEM experiments, entire litters were either left undisturbed or sleep deprived. While the behavior was monitored continuously, the behavioral scoring of each pup in the litter was too difficult to perform.

#### Adolescent (P30) mice

B6.Cg-Tg(Thy1-YFP)16Jrs/J mice were maintained on a 12/12 h light/dark cycle (lights on at 8 A.M.) with food and water available *ad libitum*. CSR (*n* = 3 mice; 2 males) started at P26 (day 1) at the beginning of the light phase and ended on day 5 (P30) 6 h after lights on. The mice were constantly monitored by direct visual inspection and with infrared cameras during the entire duration of the experiment. During the light period animals were kept awake using a combination of stimuli, including exposure to novel objects and a running wheel, social interactions with their siblings, and by changing cages and bedding. Room lights were also turned off for brief periods in the first 6 h of the light period (∼20 min/h for a total of 2 h) to provide an additional arousal stimulus and prevent the occurrence of NREM sleep. In previous studies (Alföldi et al., 1991; Benca et al., 1998; Miller et al., 1998), short periods of darkness during the light phase had no major effects on the daily amounts and circadian patterns of sleep and wake, while acutely promoted REM sleep and wake at the expenses of NREM sleep. Note that the promoting effects on REM sleep occurred in rats that could sleep *ad libitum*, while our mice were forced to stay awake. Every night, our mice were housed for several hours on a conveyer over water apparatus that runs constantly at slow speed; this apparatus allows the animals to sleep for brief periods (<8 s) before being forced to move or fall into the water. This CSR method was selected because, by relying on different types of stimuli, is effective and enforces wake in a realistic manner. Specifically, in a previous study using mice implanted for chronic EEG recordings we found that this paradigm of CSR reduces total sleep time across the 4 d by ∼70% (first day 80%; last day 63%), including a decrease of 68% in NREM sleep and of 79% in REM sleep relative to baseline (de Vivo et al., 2016). In the current study, as well as in previous ultrastructural studies (de Vivo et al., 2017), the mice were not implanted with EEG electrodes to avoid possible damage to the cortex and risk of inflammation. Instead, we relied on behavior, the presence of locomotor activity, and the ability to respond to a stimulus to assess the effectiveness of the CSR protocol. While behavior was continuously monitored, a behavioral scoring was not performed, because of the difficulty of following simultaneously several CSR mice. The CSR mice were compared with mice of the same age (P30, of either sex) killed after 6–7 h of spontaneous S or sleep deprivation (EW) during the day, and 6–7 h of SW at night (four mice/group). Behavioral states in these mice were determined by continuous monitoring with infrared cameras and motor activity was quantified by custom-made video-based motion detection algorithms, as previously detailed (de Vivo et al., 2017). We previously found that video-monitoring consistently estimates total sleep time with at least 90% accuracy, although it cannot distinguish NREM sleep from REM sleep (Maret et al., 2011). SW mice were euthanized during the dark phase (∼2–3:30 A.M.) after a period of wake of at least 1 h, interrupted by periods of sleep of <5 min, and after spending at least 70% of the previous 6–7 h awake. S mice were euthanized during the light period (∼2–4:45 P.M.), after a long period of sleep of >45 min, interrupted by periods of wake of <4 min, and after spending at least 75% of the previous 6–7 h asleep. EW mice were kept awake during the first part of the light period (6–7 h) by exposure to novel objects and running wheel and by tapping on the cage whenever the animals appeared drowsy. The changes in ASI size in the M1 and primary sensory cortex of S, EW and SW mice have been published before (de Vivo et al., 2017), the data from M1 of these animals are shown here to provide a reference against which to compare the results in the CSR group. All four groups of mice (CSR, S, EW, SW) were collected at the same time and brain samples were processed and imaged using serial electron microscopy within the same period of a few months, alternating between mice of the different experimental groups. The initial segmentation of the spines for the CSR group was also done concurrently with that of the other P30 mice by the same trained annotators, who were blind to experimental condition. Because of the time-consuming nature of manual segmentation, however, and the decision to focus first on the results pertaining the normal sleep/wake cycle and short sleep deprivation conditions (de Vivo et al., 2017), the final analysis of the CSR mice was postponed and completed only for the current study. For the final analysis, as in the previous study (de Vivo et al., 2017), all segmentation data were further tested for accuracy and consistency by the same experienced tracer (L.d.V.).

### Experiments to assess weight loss during sleep deprivation

B6.Cg-Tg(Thy1-YFP)16Jrs/J mice were maintained on a 12/12 h light/dark cycle (lights on at 8 A.M.) with food and water available *ad libitum*. Mice were inspected every day for new pups and P0 was defined as the day of birth. At P12, each mouse (of either sex) was marked on its tail and weighed. At P13, mice were assigned to two experimental groups: in the control group pups were kept with their dam and left undisturbed during the experiment. In the sleep deprivation group, pups were kept with their dam but were deprived of sleep by gentle handling and novel objects for 16 h from zeitgeber time (ZT)0 to ZT16. To monitor the body weight of the pups, we weighed them as frequently as possible while trying not to disturb their sleep/wake behavior. Specifically, at P13 the sleep deprived pups were weighted every 4 h, while the control mice were weighted only at ZT0 and ZT16. To monitor the recovery from the weight loss, we also weighed all pups at ZT10 on the day after sleep deprivation.

### Tissue preparation for electron microscopy and image acquisition

Methods have been described in detail in previous studies (de Vivo et al., 2017, 2019). Blocks of tissue (1 mm^2^) encompassing the M1 (1.85 mm anterior to bregma, 1.5 mm lateral) were stained blind to experimental condition and serial images were obtained using a ΣIGMA VP field emission scanning electron microscope (Carl Zeiss NTS Ltd) equipped with 3View technology (Gatan Inc.) and a backscattered electron detector (aperture of 30 μm, high vacuum, acceleration voltage of 1.5–2 kV, image size of 5000 × 5000 pixels, image resolution in the *xy*-plane of 4 nm). Nominal thickness of the ultrathin sections was 40–50 nm. Stacks of ∼500 images each were acquired per mouse (∼10,000–21,000 μm^3^) in layer 2 of M1, as in the previous studies (de Vivo et al., 2017, 2019). Images were Gaussian filtered and automatically aligned using the open source software Fiji (Schindelin et al., 2012). Average actual section thickness for each stack was estimated using the cylindrical diameters method as previously described (Fiala and Harris, 2001), and was comparable across conditions. Dendritic segments and all their protrusions were segmented manually in TrakEM2 (Cardona et al., 2012) by trained annotators who were blind to experimental condition. We randomly selected spiny dendritic segments whose diameter ranged between 0.44 and 2.24 μm (P13) and between 0.54 and 1.74 μm (P30). Densely spiny dendrites located in layer 2 include intermediate and terminal dendrites of layer 3 and 5 pyramidal neurons, as well as basal and oblique dendrites of layer 2 pyramidal neurons (Larkman, 1991; van Aerde and Feldmeyer, 2015). To focus on pyramidal cells, we excluded dendritic segments with few or no spines, as well as dendrites with few spines and whose number of shaft synapses exceeded the number of spine synapses.

As described before (de Vivo et al., 2017), all protrusions were defined as “spines” (in agreement with Holtmaat and Svoboda, 2009), and distinguished in spines with or without synapses. Criteria to define a synapse included the presence of a presynaptic bouton with at least one to two synaptic vesicles within a 50-nm distance from the cellular membrane facing the spine, a visible synaptic cleft and a postsynaptic density. Distribution of dendritic diameters was balanced across experimental groups (P13, *p* = 0.45; P30, *p* = 0.15). In each spine the presence/absence of the following structures was recorded: spine apparatus, one or more spinulae in the head or neck of the spine, and mitochondria in the dendritic shaft close to the spine neck and in the axonal bouton. In almost all cases a mitochondrion was present along the dendritic shaft in the vicinity of the spine neck, while mitochondria were almost never found inside the spine head. The presence of components of the non-smooth endoplasmic reticulum (non-SER), classified according to Cooney et al. (2002), was also recorded. The non-SER elements include tubules, small uncoated vesicles, large coated or uncoated vesicles, and multivesicular bodies (MVBs), and are distinct from the continuous network of SER present throughout the dendritic shaft (Cooney et al., 2002).

As described before (de Vivo et al., 2017, 2019), the ASI was used as the ultrastructural measure of synaptic strength and traced at the interface between the spine head and the presynaptic terminal or bouton. We focused on the ASI because in SBEM images its exact borders are easier to identify than those of the postsynaptic density (Holcomb et al., 2013).

Although the ASI is a less established measure than the postsynaptic density, its size correlates with the size of the postsynaptic density and of the spine head (Cheetham et al., 2014), which in turn correlate with the synaptic expression of glutamatergic AMPA receptors and with the amplitude of synaptic excitatory currents (Nusser et al., 1998; Matsuzaki et al., 2001; Katz et al., 2009).

Specifically, the region of contact between the two apposed objects was outlined in each individual section using the arealist brush suitably set at 1 pixel size. In this way, a quasi two-dimensional sheet-like object representing the interfaced region was created along the z dimension. The total surface area was calculated by computing the smoothed upper bound surface, according to the formula:

Smoothed upper boundsurface=∑k=0n(Ps(a)×12T) + (Ps(b)×12T) + [A(a)−A(b)],

where n is the number of sections, a and b are the traced elements at the top and bottom of a section k of thickness T, Ps is the smoothed perimeter, and A is the area (Cardona et al., 2012). Finally, the areas of the traced element in the section k = 1 and in the section k = n were subtracted from the smoothed upper bound surface value and the result was divided by 2 to get an approximate value of the apposed surface.

### Statistical analysis

Statistical analysis was performed using linear mixed effects (LMEs) models that include both random and fixed effects. The random effects are used to account for the fact that multiple spines are sampled from the same mouse and dendrite. LME models offer several advantages over repeated measures ANOVA including the ability to handle unbalanced designs, which is the case in this study.

The general form of these models is

y=Zu + Xβ + ϵ,

where

u ∼ G(0,Σ),

and

$$\text{ϵ}\;∼\;\text{G}\left(0,{\text{σ}}^{2}\text{I}\right)$$

In these models, y is a vector of response variables (typically log(ASI) values), u is a vector of random effects (mouse and dendrite effects), β is a vector of fixed effects, X and Z are design matrices that link the response variable to the random and fixed effects, and ϵ is a vector of residual values. Both the random effects (u) and the residuals (ϵ) are assumed to have a Gaussian distribution, while the residuals are additionally assumed to be independent with constant variance. Model assumptions of normality and constant variance were assessed and validated graphically using residual plots for all models presented.

For response variables measured at the dendrite level (spine density, dendrite diameter), experimental condition was the only fixed effect included in the models, and mouse was the only random effect. For models at the synapse level (ASI, and interactions with the various organelles), we included dendrite as a potential random effect, and dendrite diameter as a fixed effect. Maximum likelihood estimates of the parameters in the LME models were calculated numerically using the *lmer*() function of the *lme4* package in R (Bates et al., 2015), and the statistical significance of effects was assessed using likelihood ratio tests. For significant effects of condition, pairwise *post hoc* tests with *p* values corrected for multiple comparisons were performed using the *glht*() function in the *multcomp* package (Bretz et al., 2011).

### LME models

The parameter estimates for the LME models (P30: ASI; P13: ASI and synapse density) are shown in Table 1. A log transformation was applied to ASI values and a square-root transformation was applied to spine density values to stabilize variance and normalize residuals. The assumption of normally distributed residual values with constant variance was assessed graphically in the resulting models. Based on a scatter plot of the estimated residuals versus fitted values, and a quantile-quantile plot of the estimated residuals, we found no evidence against these assumptions.

**Table 1:** Parameters for LME models

P30 data: ASI model (log transformation)			
Random effects	SE		
Dendrite (intercept)	0.0792		
Mouse (intercept)	0.0705		
Residual	0.9470		
Fixed effects	Level	Estimate	SE
Intercept		−1.9129	0.0873
Dendrite diameter	Continuous (linear)	0.1818	0.0718
Condition	CSR (reference)	0	0
	EW	−0.0902	0.0894
	SW	−0.1751	0.0864
	S	−0.1166	0.0845
Endosome		0.1985	0.0644
Condition × endosome	CSR (reference)	0	0
	EW	0.0781	0.0874
	SW	0.2596	0.0840
	S	−0.1284	0.0831
			
P13 data: ASI model (log transformation)			
Random effects	SE		
Dendrite (intercept)	0.1025		
Mouse (intercept)	0.0273		
Residual	0.8974		
Fixed effects	Level	Estimate	SE
Intercept		−2.1445	0.0566
Dendrite diameter	Continuous (linear)	0.1656	0.0522
Condition	EW15 (reference)	0	0
	EW4	0.3666	0.0483
	S	0.2317	0.0460
	Rec	−0.1179	0.0503
			
P13 data: density model (spines with synapse; square root transformation)			
Random effects	SE		
Mouse (intercept)	0.0380		
Residual	0.1217		
Fixed effects	Level	Estimate	SE
Intercept		0.6077	0.0239
Condition	EW15 (reference)	0	0
	EW4	−0.1032	0.0369
	S	−0.0559	0.0347
	Rec	−0.0344	0.0384

Scaling analysis. To assess the presence of a scaling relationship, we tested the hypothesis that H_0_: f_1_(x) = f_2_(x – c), i.e., that two probability distributions on a log scale differ only by a location shift. We first estimated c as the difference in the median of the two distributions, and then transformed the data so that under the null hypothesis they will have the same distribution. Finally, we applied the Kolmogorov–Smirnov test for equality of distribution between two samples.

## Results

### Spines with endosomes have larger ASI after CSR than after sleep in one-month-old mice

**Figure 1.**
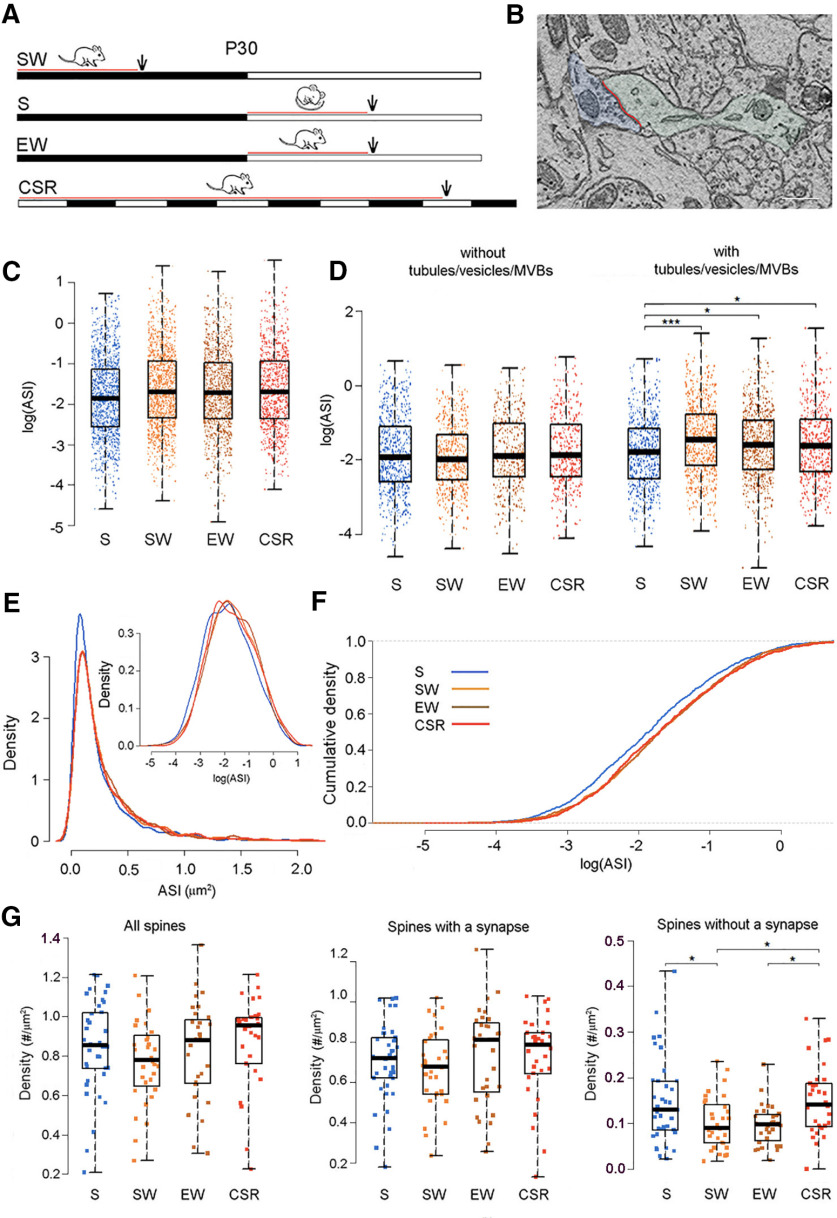
Sleep/waking changes in ASI size in one-month-old mice. ***A***, Experimental groups. The black and white horizontal bars indicate the dark and the light period, respectively. The red lines indicate the time window used to select the mice based on their sleep/waking behavior, and the arrows show when the brains were collected. ***B***, Raw image of a spine containing a synapse (spine and dendrite in light green, ASI in red; axonal bouton in light blue). Scale bar: 300 nm. ***C***, The size of the ASI in all measured synapses, each synapse represented by one dot: sleep (blue), SW (orange), enforced wake (EW; brown), and CSR (red). ***D***, as in ***C***, but with synapses divided based on the presence or absence of components of the non-SER, including tubules, vesicles, and MVBs. *, S < CSR, *p* = 0.0235; *, S < EW, *p* = 0.0175; ***, S < SW, *p* = 0.0001. ***E***, Log-normal distribution of ASI sizes in the four experimental groups; top right, same on a log scale. ***F***, Cumulative plots of ASI sizes in all groups. ***G***, Density (*N* per μm^2^ of surface area) of all spines, and separately for spines with and without a synapse. Each dot is one dendrite. *, SW < **S**, *p* = 0.0448; *, EW < CSR, *p* = 0.0448; *, SW < CSR, *p* = 0.0354.

We previously reported that, in layer 2 of the M1 and primary sensory cortex, the size of the ASI in axospinous excitatory synapses is larger after 6–7 h of SW or sleep deprivation (EW) as compared with 6–7 h of S (de Vivo et al., 2017). Here, we measured the ASI in mice CSR for >4 d (102 h, three CSR mice, 905 spines with synapse), and compared these results with the previously published data from the M1 of S, SW, and EW mice (Table 2; Fig. 1*A*,*B*). When the brains were collected, all mice were 30 d old.

**Table 2:** Summary of ultrastructural measures

	P30				P13			
	S	SW	EW	CSR	S	EW4	EW15	Rec
Total *N* of dendrites	38	32	31	31	57	51	54	36
Total *N* of measured spines with synapse	1367	1347	1167	905	1066	912	1398	842
ASI (μm^2^, mean ± std) Range (μm^2^)	0.26 ± 0.29 0.01–2.04	0.30 ± 0.34 0.01–4.02	0.29 ± 0.32 0.01–3.54	0.31 ± 0.37 0.02–4.61	0.25 ± 0.26 0.01–2.17	0.30 ± 0.34 0.01–2.60	0.20 ± 0.23 0.01–2.33	0.19 ± 0.23 0.01–2.01
Spine density (all protrusions) (#/μm^2^, mean ± std)	0.85 ± 0.24	0.77 ± 0.21	0.82 ± 0.25	0.88 ± 0.23	0.40 ± 0.16	0.35 ± 0.13	0.49 ± 0.16	0.46 ± 0.24
Spine density (spines with synapse; #/μm^2^, mean ± std)	0.70 ± 0.20	0.67 ± 0.19	0.73 ± 0.24	0.73 ± 0.22	0.31 ± 0.13	0.25 ± 0.10	0.38 ± 0.15	0.36 ± 0.21
Spine density (spines without synapse; #/μm^2^, mean ± std)	0.15 ± 0.09	0.10 ± 0.06	0.10 ± 0.04	0.15 ± 0.08	0.09 ± 0.05	0.10 ± 0.06	0.10 ± 0.04	0.10 ± 0.06
Spines without synapse (%, mean ± std)	16.0 ± 2.8	12.7 ± 3.3	11.9 ± 3.2	17.4 ± 3.5	22.0 ± 5.4	26.3 ± 4.4	21.8 ± 4.1	22.6 ± 5.2
Dendrite diameter (μm, mean ± std)	0.87 ± 0.23	0.92 ± 0.24	0.84 ± 0.22	0.78 ± 0.18	0.92 ± 0.33	0.94 ± 0.35	0.90 ± 0.24	1.00 ± 0.30
Dendrite length (μm, mean ± std)	21.9 ± 7.0	26.2 ± 4.7	22.8 ± 5.9	19.3 ± 5.1	23.4 ± 8.5	26.3 ± 7.1	26.7 ± 8.7	25.0 ± 5.7
Spines with spine apparatus (%, mean ± std)	26.1 ± 4.3	30.5 ± 4.5	27.1 ± 3.1	30.2 ± 3.5	7.4 ± 2.5	7.2 ± 1.9	5.4 ± 2.3	9.4 ± 3.2
Spines with non-SER elements (tubules/ vesicles; %, mean ± std)	49.7 ± 7.0	56.3 ± 13.0	61.2 ± 6.8	54.4 ± 6.2	70.1 ± 4.2	74.6 ± 7.1	72.6 ± 3.9	66.0 ± 8.3

All protrusions are defined as spines. In oblique spines, the ASI could not be measured because the synapse was oriented obliquely or orthogonally to the cutting plane. std, standard deviation.

We applied an LME model that uses sleep/wake condition as categorical fixed effect, dendrite diameter as linear fixed effect, and mouse and dendrite as random effects (see Materials and Methods). We found a trend main effect of condition (*p* = 0.0568) and *post hoc* tests revealed a significant increase in ASI size in the CSR mice as compared with the S mice (*p* = 0.0466) with no difference between CSR and the other wake groups (EW vs CSR, *p* = 0.9865; SW vs CSR, *p* = 0.9874; Fig. 1*C*). We previously found that the sleep/wake changes in ASI size occur specifically in spines containing vesicles, tubules, coated vesicles and/or MVBs, which together represented the majority of the spines (de Vivo et al., 2017). These organelles are believed to be mostly of endosomal origin (Cooney et al., 2002) and for brevity we refer to them here as “endosomes.” In line with the previous results (de Vivo et al., 2017), we found a significant difference between S and CSR mice in the spines containing endosomes. Specifically, using an LME model with condition, endosome (binary), dendrite diameter and condition × endosome interaction, we found a significant interaction between condition and endosome (*p* = 5.1e-6). *Post hoc* tests revealed that for synapses with endosomes, the ASI size was significantly smaller in the S group relative to all wake groups, including the CSR group (S < CSR, *p* = 0.0235; S < EW, *p* = 0.0175; S < SW, *p* = 0.0001; Fig. 1*D*). The three wake groups did not differ from each other (EW = CSR, *p* = 0.9999; SW = CSR, *p* = 0.8998; SW = EW, *p* = 0.7681). There were no significant differences between any conditions for the synapses without endosomes. The distribution of ASI sizes in the CSR mice was log-normal, as reported in the other groups (de Vivo et al., 2017; Fig. 1*E*). The cumulative distribution of ASI sizes in the S group was shifted to the left relative to all other groups, including the CSR mice, whose curve overlapped completely with those of the W and SD mice (Fig. 1*F*). Formal testing confirmed that, in line with the presence of scaling in S mice relative to EW and SW mice (de Vivo et al., 2017), the decrease in ASI size in S mice relative to CSR mice also obeyed a scaling relationship (no evidence against a scaling relationship between S and CSR; *p* = 0.9414). The percentage of spines that contained tubules and vesicles or a spine apparatus, did not change across groups (Table 2). The density of the spines with a synapse did not change across groups (*p* = 0.8315), but there was a significant difference in the density of spines without a synapse (*p* = 0.0261). *Post hoc* tests revealed that the density of spines without a synapse was significantly higher in CSR relative to both the EW and SW mice, and higher in S relative to SW mice (EW < CSR, *p* = 0.0448; SW < CSR, *p* = 0.0354; S = CSR, *p* = 0.9953; EW < S, *p* = 0.0564; SW = EW, *p* = 0.9999; SW < S, *p* = 0.0448; Fig. 1*G*). Thus, after CSR the mean ASI size is comparable to that measured after SW or short sleep deprivation, and larger than the mean ASI size after sleep.

### EW in young pups leads to a decrease in ASI size

**Figure 2.**
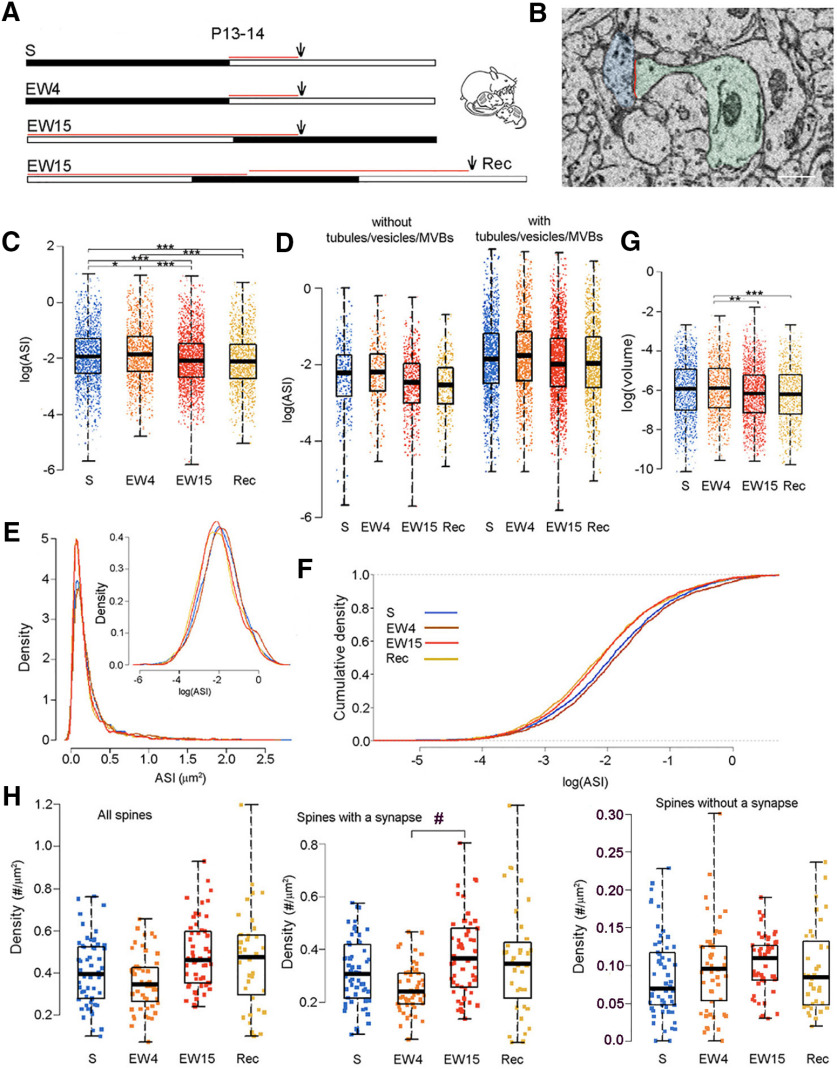
Sleep/wake changes in ASI size in two-week-old mouse pups. ***A***, Experimental groups. The black and white horizontal bars indicate the dark and the light period, respectively. The red lines indicate the time window used to select the mice based on their sleep/waking behavior and the arrows show when the brains were collected. ***B***, Raw image of a spine containing a synapse (spine and dendrite in light green, ASI in red; axonal bouton in light blue). Scale bar: 300 nm. ***C***, The size of the ASI in all measured synapses, each synapse represented by one dot: sleep (blue), SW (orange), enforced wake (EW; brown), and CSR (red). *, EW4 > S, *p* = 0.0368; ***, EW15 < EW4, *p* = 7.2e-14; ***, EW15 < S, *p* = 2.1e-6; ***, Rec < EW4, p < 2.2e-16; ***, Rec < S, *p* = 7.6e-11. ***D***, as in ***C***, but with synapses divided based on the presence or absence of components of the non-SER, including tubules, vesicles, and MVBs. ***E***, Log-normal distribution of ASI sizes in the four experimental groups; top right, same on a log scale. ***F***, Cumulative plots of ASI sizes in all groups. ***G***, The endosome volume in all spines containing tubules, vesicles, and MVBs. Each dot represents one spine. **, EW15 < EW4, *p* = 0.0022; ***, Rec < EW4, *p* = 0.0004. ***H***, Density (*N* per μm^2^ of dendrite surface area) of all spines, and separately for spines with and without a synapse. Each dot is one dendrite. #, EW15 > EW4, *p* = 0.026 after trend effect of condition *p* = 0.0896.

In P13 pups, we previously found in layer 2 of M1 that the ASI size of axospinous excitatory synapses is larger after 4–5 h of sleep deprivation (EW) as compared with 4–5 h of S (de Vivo et al., 2019). Here, we used four new groups of pups aged approximately two weeks (Fig. 2*A*). Three experimental groups were collected at P13: S pups could sleep during the first 4–5 h of the light period (*n* = 4); EW4 pups were kept awake as much as possible for 4–5 h at the same time of day (*n* = 3); EW15 pups were forced to stay awake as much as possible for 15–16 h starting at light onset on P13 (*n* = 4). A fourth group of pups was sleep deprived for ∼15 h starting at the beginning of the light phase at P13, then allowed to recover sleep *ad libitum* 15–16 h until the afternoon of P14 (recovery, Rec; *n* = 3). Overall, we analyzed ∼281 spines per mouse (range 260–328) for a total of 1066 spines in S, 912 in EW4, 1398 in EW15, and 842 in Rec (Fig. 2*B*).

We applied an LME model that uses sleep/wake condition as categorical fixed effect, dendrite diameter as linear fixed effect, and mouse and dendrite as random effects (see Materials and Methods). We found a main effect of condition (*p* = 8.7e-7) and *post hoc* tests revealed that the ASI size increased after a few hours of sleep deprivation (EW4) as compared with S (*p* = 0.0368), consistent with our previous findings (de Vivo et al., 2019). By contrast, the size of the ASI decreased after longer sleep deprivation (EW15) relative to both EW4 and S (EW15 < EW4, *p* = 7.2e-14; EW15 < S, *p* = 2.1e-6; Fig. 2*C*). The ASI size did not change significantly after recovery from long sleep deprivation, that is, it did not differ between Rec and EW15 pups and was still significantly smaller in Rec pups than in S and EW4 pups (Rec < EW4, *p* < 2.2e-16; Rec < S, *p* = 7.6e-11; Fig. 2*C*). By applying an LME model with condition, endosome (binary), dendrite diameter, and condition × endosome interaction, we found no significant interaction between condition and endosome (*p* = 0.8644), indicating that the decrease in ASI size with EW was not restricted to spines with endosomes (Fig. 2*D*). The distribution of ASI sizes in the pups was log-normal, as previously reported (de Vivo et al., 2019; Fig. 1*E*) and the cumulative plots of ASI sizes showed a clear shift to the left in the EW15 and Rec pups relative to the S and EW4 pups, as well as a shift to the left in S pups relative to EW4 pups (Fig. 2*F*). Formal testing confirmed the presence of scaling between S mice and EW15 mice (no evidence against a scaling relationship between S and EW15; *p* = 0.8446). The percentage of spines that contained tubules and vesicles, or a spine apparatus, did not change across groups (Table 2), nor did the volume of the spine apparatus (*p* = 0.117; LME model with condition and diameter as fixed effects, mouse and dendrite as random effects). We found a significant effect of condition on tubules/vesicles volume (*p* = 0.0068; LME model with condition and diameter as fixed effects, mouse and dendrite as random effects). *Post hoc* tests revealed that the tubules/vesicles volume was significantly lower in EW15 and Rec pups relative to EW4 pups (EW15 < EW4, *p* = 0.0022; Rec < EW4, *p* = 0.0004; Fig. 2*G*). A trend decrease in tubules/vesicles volume was also present in S pups relative to EW4 pups (*p* = 0.0803). The total density of the spines (with and without a synapse) did not change across groups (*p*= 0.1171; LME model with condition as a fixed effect and mouse as a random effect). The density of the spines with a synapse, however, showed a trend effect of condition (*p* = 0.0896) and *post hoc* tests showed a significant increase in EW15 relative to EW4 pups (*p* = 0.0261; Fig. 2*H*). There was no significant effect of condition on the spines without a synapse (*p* = 0.7458). The synapses of EW15 pups had smaller ASI but were more numerous than in EW4 pups. Since newly formed synapses are on average small, we tested whether the difference in ASI size between the two groups could be accounted for by the smallest spines. This was not the case: by fitting an LME model with condition (EW4 and EW15 only) and diameter as fixed effects, and mouse/dendrite as random effects, we found that the difference in ASI size between EW15 and EW4 pups became insignificant only after removing the 60% smallest synapses. Thus, in young pups short and long sleep deprivation have opposite effects on the ASI size, the former increasing it and the latter decreasing it relative to sleep, and this difference is unlikely to be because of the conversion of small protrusions without a synapse into spines with a synapse.

**Figure 3.**
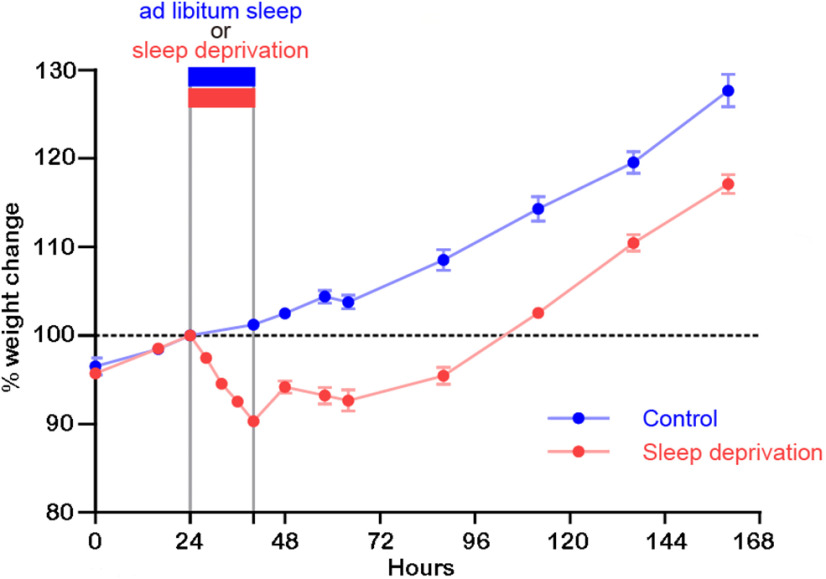
Weight changes before, during and after 16 h of sleep deprivation in two-week-old mouse pups. Weight change in pups sleep deprived for 16 h (*n* = 10) or left undisturbed (*n* = 12). Weight change is shown relative to hour 24 (100%).

To test whether long sleep deprivation results in a significant weight loss, two additional groups of P13 pups were studied: control pups were kept with their dam and left undisturbed during the experiment, while sleep deprived pups were kept with their dam and sleep deprived for 16 h as in the experiment described above. While control pups maintained their weight during the 16 h of the experiment, the sleep deprived pups lost on average 10% of their weight (Fig. 3). Body weight increased in these pups after the experiment ended, but remained below the values in controls 5 d afterward (increase relative to the day before sleep deprivation; Control mice = 27.7 ± 1.8%, sleep-deprived mice = 17.1 ± 1.1%).

## Discussion

In a first series of experiments, we studied one-month-old YFP mice. At this age both the circadian and the homeostatic regulation of sleep are present and the sleep of these adolescent animals is similar, albeit not identical, to that of adult mice (Nelson et al., 2013). CSR was enforced using a combination of exposure to novelty, social interactions, access to a running wheel, and other stimuli. This method was previously characterized in our laboratory using polygraphic recordings (de Vivo et al., 2016). Specifically we found that in the fourth week of life, with this paradigm, sleep is reduced to approximately a third of the normal daily amount and both NREM and REM sleep are decreased (68% decrease in NREM sleep and 79% decrease in REM sleep over 4 d; de Vivo et al., 2016). Sleep episodes occur throughout the 24-h cycle, either when the mice escape the attention of the experimenter or when they are allowed to sleep for 10–20 min because they appear too tired. Thus, with this paradigm short bouts of sleep occur day and night, and both sleep phases are decreased by at least two/thirds. Few other studies have examined the effects of chronic sleep loss during development. Several experiments in cats and rodents have used chronic deprivation of REM sleep (7–10 d) around the critical period and demonstrated that this manipulation disrupts the maturation of the visual pathways (Oksenberg et al., 1996; Hogan et al., 2001; Shaffery et al., 2006). In prairie voles, one week of sleep disruption during the third week of life increases the number of inhibitory cells in the primary sensory cortex and impairs social bonding behavior in adulthood (Jones et al., 2019). The cage-shaking method used in prairie voles results in fragmented sleep and a significant decrease in REM sleep, but there were no effects on NREM sleep (Jones et al., 2019). Other studies in adolescent mice have used total or selective sleep deprivation, but only for a few hours (Maret et al., 2011; Li et al., 2017). In short, it is difficult to directly compare our results with previous findings, because the extent and the pattern of sleep disruption vary significantly across studies.

We found that in one-month YFP-H mice, the majority of cortical synapses in the superficial layers of M1, those containing endosomes, have larger ASIs after >4 d of sleep restriction than after sleep, and as large as after several hours of either SW or short sleep deprivation. Thus, the severely curtailed sleep of CSR mice was not enough for synaptic renormalization to occur, at least in terms of structural changes in ASI. It remains possible that the short episodes of sleep were enough to decrease other measures of synaptic strength that can change more rapidly, for instance the phosphorylation levels of GluA1-containing AMPA receptors at Ser831 and Ser845 (Vyazovskiy et al., 2008; Hinard et al., 2012; Suzuki et al., 2020). However, synaptic weakening ultimately involves the endocytosis of the AMPA receptors from the synaptic surface. This process can happen within a few hours and the synaptic expression of GluA1-containing and GluA2-containing AMPA receptors is indeed lower after 4–8 h of sleep than after 4–8 h of wake (Vyazovskiy et al., 2008; Diering et al., 2017). Because the synaptic levels of AMPA receptors correlate with synaptic size (Nusser et al., 1998; Zhang et al., 2015; El-Boustani et al., 2018), it is unlikely that the expression of these receptors in the spine could decline without a decline in ASI size. Thus, our results suggest that sleep-dependent synaptic renormalization is incompatible with the severity of the sleep loss experienced by the chronically restricted mice. We cannot be sure that other CSR methods would have produced the same outcome, nor can we rule out that the CSR method that we used, or any other method, may have confounding effects on the synapses unrelated to sleep loss per se.

The ASI size, and several other features of the organelles inside the spines, did not differ in the CSR mice relative to the other wake groups. Based on these parameters, one would have to conclude that severe CSR does not affect synapses and spines above and beyond what normal wake or a short period of sleep deprivation does. However, we previously obtained ultrastructural evidence that CSR puts a greater burden on pyramidal excitatory neurons than acute sleep deprivation. In mice of the same strain and age, and in the same region studied in the current study (layer 2 of M1), we found that the percent of cytoplasm occupied by mitochondria is larger after CSR than after sleep, and at intermediate levels after short sleep deprivation (de Vivo et al., 2016). The density of secondary lysosomes, which digest damaged cellular components, is also significantly higher after CSR relative to sleep, with no difference between sleep and short sleep deprivation (de Vivo et al., 2016). In another study, we found that in the same cortical area >80% of the spine heads, all but the smallest spines, are contacted by peripheral astrocytic processes and the extent of this astrocytic coverage increases with CSR relative to both sleep and short sleep deprivation (Bellesi et al., 2015). The percent of the ASI perimeter directly contacted by astrocytes also increases with CSR relative to both sleep and short sleep deprivation (Bellesi et al., 2015). Together, these results suggest that the pyramidal excitatory neurons require more mitochondria during CSR and rely more extensively on the peripheral astrocytic processes, presumably for the clearance of glutamate and potassium ions from the synaptic cleft. Thus, after CSR the axospinous synapses may be similar in size to those after normal wake, but the cellular burden to maintain them may be higher. Of note, in a previous study we also found that, although all mice gained weight between P21 (weaning) and P30 (end of CSR), controls did so more than CSR mice (Billeh et al., 2015), suggesting that even when food is available *ad libitum*, adolescent mice struggle to maintain the energy balance during severe sleep loss.

In a previous study employing a similar protocol of chronic sleep loss during early adolescence (P25–P30), we found some evidence for long-term effects on brain connectivity (Billeh et al., 2015). In that study, we focused on the secondary motor cortex, a “higher order” area with widespread projections and whose functional maturation continues throughout adolescence. In adult mice, more than a month after the end of the sleep restriction period, we measured the projection fraction, a combined measure of passing fibers and terminals originating from the secondary motor cortex. Although the results were subtle and heterogenous across animals, this measure of connectivity showed an overall decrease in adult mice previously sleep restricted relative to their siblings, and machine learning algorithms could classify significantly above chance the two groups. These findings suggest that structural connectivity may have been affected in the long term, perhaps as a consequence of the cellular burden demonstrated by the experiments mentioned above.

In a second series of experiments, we found that at P13 the ASI size was affected in opposite directions by short and long sleep loss: larger ASI size were present after 4 h of sleep deprivation compared with sleep, consistent with the results in P30 mice, while 15 h of forced wake led to a decrease in ASI size relative to both sleep and short sleep deprivation. These effects were not associated with significant changes in the number of spines, but there was a trend for the density of spines with synapses to be higher after 15 h than after 4 h of sleep deprivation. Synaptogenesis and synaptic pruning in rodents occur mainly during the second postnatal week (Cirelli and Tononi, 2015). In line with this, in the same mouse line used in the current study (YFP-H) we previously found that the cortical levels of Synapsin I, a ubiquitous presynaptic marker, increase greatly from P10 to P20 and slightly from P20 to P25, but are stable afterward (de Vivo et al., 2014). Thus, it may not be surprising that sustained wake at P13, even if “just” for 15 h, had synaptic effects that went beyond those seen after shorter periods of wake, while CSR at P30 did not.

A possible explanation for the small ASI sizes after 15 h of sleep deprivation is that despite our efforts to keep them awake, the pups were actually asleep most of the time, especially after the first few hours of sleep deprivation. Indeed, in this study we did not attempt to score sleep and wake states in 30-s epochs using behavioral criteria, as we previously did (de Vivo et al., 2019). However, after 15 h of sleep deprivation the ASI size was even smaller than after 4 h of sleep, and after 16 h of recovery it remained smaller than after normal sleep. Because the density of synapses showed a trend to increase from 4 to 15 h of sleep deprivation while it did not differ between 4 h of sleep and 4 h of EW, it is also possible that EW15 synapses were small because many of them were newly formed. However, we found no evidence to support this hypothesis, because the average ASI size remained significantly smaller in EW15 than in EW4 pups until at least 60% of the smallest synapses were removed from the comparison. Finally, the overall shrinkage of ASI size with long sleep deprivation may indicate that the immature brain can meet the high demand of sustained synaptic activity for a limited amount of time, in our case 4 h. Synaptic activity accounts for the bulk of the brain’s energy need (Attwell and Laughlin, 2001; Karbowski, 2014), and to grow and survive, synaptic connections require continuous protein synthesis (Kleim et al., 2003) and a steady supply of energy (Li et al., 2004). In flies, 36 h of sleep deprivation starting soon after eclosion impair the development of one specific olfactory glomerulus, the one that normally shows the largest growth during the same period (Kayser et al., 2014).

In a previous study, we found that P13 pups struggle to eat while sleep deprived (de Vivo et al., 2019). In the current study, despite our best attempts to promote feeding, we found that sleep deprived pups lost weight during the experiment. The increased energy need caused by enriched and prolonged wake, combined with suboptimal access to food, may have contributed to the decrease in ASI size. The inevitable stress caused by the stimulation used to enforce wake may also have contributed. While we tried to limit the stress by keeping the litter with the dam in their home cage, maternal factors (grooming, feeding and warmth) were likely affected by the stimulation. Thus, sleep deprivation may have resulted in high glucocorticoid or mineralocorticoid levels, and/or high catecholamine levels, although a reduced capacity to release corticosterone is well documented in rodent pups during the first two weeks of life (Varga et al., 2013). Future experiments should determine to which extent these signaling pathways can account for the different synaptic effects of four compared with 15 h of sleep deprivation (Popoli et al., 2011). Of note, the decrease in ASI size was still present 16 h after the end of sleep deprivation and, 5 d later, body weight was still below control levels. While we did not measure the ASI size at this later time point, these results suggest that when body and brain are growing at high speed, the recovery from prolonged sleep deprivation may take a long time, or may not be complete.

## Synthesis

Reviewing Editor: Rae Silver, Columbia University Decisions are customarily a result of the Reviewing Editor and the peer reviewers coming together and discussing their recommendations until a consensus is reached. When revisions are invited, a fact-based synthesis statement explaining their decision and outlining what is needed to prepare a revision will be listed below. The following reviewer(s) agreed to reveal their identity: Radhika Basheer.

Major comments:

Most problematic is the use of prior work for controls: The dataset for long sleep deprivation in older (P30) mice is incomplete. The authors perform long sleep deprivation but do not provide matched control data. Instead they compare the results to their earlier published work. They must have matched control data to conclude anything from these studies. Basically, the P30 data cannot published without the controls. It is not possible to compare one condition collected now with 3 conditions collected and published 6 years ago by different experimenters, on mouse colonies in which there may have been considerable genetic background changes over the ensuing years. The use of controls for the present study requires a great deal of new work. Perhaps the data on young animals stands on its own.

There appear to be confounding effects of light pulses: One concern is in the method of CSR. The authors describe brief interruptions in the light by switching off the light for arousal of the mice. This method can introduce the confounding effects of light conditions on sleep physiology and thus the behavior. Light flashes during dark period increase REM and sleep time and vice versa. Thus, this aspect of stimulation is not acceptable for keeping the mice awake. If the authors think this method does not introduce any confounding effects on synaptic measures then it will be helpful to have a discussion on the appropriateness of this method. A better description of the timing, duration, and frequency of manipulations and how they control for things like circadian disruption by light manipulation is also needed.

There are issues regarding the sleep deprivation manipulations. It is intriguing as to how the mice were kept awake for 15h. It is generally difficult to keep the mice awake beyond 6h and much more difficult after 8h. As is also described by the authors. “As previously reported (de Vivo et al., 2019), even after the first few hours of forced wake pups responded to the stimuli much less than older mice, and it was impossible to keep them fully awake for extended periods.” The use of multiple methods in itself is problematic without appropriate controls. Furthermore, sleep deprivation is stressful. This aspect is accentuated in adolescent pups. It will be helpful to compare the changes with another stressful model (such as maternal separation 24h prior to sacrifice) to confirm the effects to be specific to sleep deprivation and not due to any stressful pathway activation.

A somewhat related problem is that there is no behavioral scoring of sleep deprivation in this study; instead the authors rely on earlier studies to support the efficacy of their deprivation paradigm. Especially given the difficulty of depriving young mice for long periods of time a verification that they have indeed succeeded in depriving them of sleep seems important.

Other comments:

The use of ASI as a method of evaluating the synaptic structure must be very well explained and clearly justified. It was unclear to reviewers how robust this measure might be.

-Reading the methods it is not clear how the state durations were evaluated.

-The description of pups experimental group is repeated both in the method and result section.

-In the abstract the Ns are misleading (adult 15 and pups 14). These are total number of mice used for each study but the N' of the experimental groups remain very small (for example in the pups study the Ns for each group is as follows: Sleep control (4) SD (3), 15 SD (4) and 15h SD+ 16 RS (3)). This should be clearly stated in the abstract.
